# Efficacy and anthropometric predictors of negative-pressure therapy for recurrent concealed penis in pediatric patients

**DOI:** 10.3389/fped.2026.1845192

**Published:** 2026-06-18

**Authors:** Shengqi Zheng, Bingqian Yin, Ruiyun Xue, Meng Fu, Hong Chen, Xu Cui, Chaoming Zhou

**Affiliations:** 1 College of Clinical Medicine for Obstetrics & Gynecology and Pediatrics, Fujian Medical University, Fuzhou, Fujian, China; 2 Department of Pediatric Surgery, Fujian Children’s Hospital (Fujian Branch of Shanghai Children’s Medical Center), Fuzhou, Fujian, China

**Keywords:** concealed penis, negative-pressure therapy, pediatric urology, postoperative recurrence, salvage intervention

## Abstract

**Background:**

The present study aimed to evaluate the clinical efficacy, safety, and three-month durability of negative-pressure therapy (NPT) as a non-surgical salvage intervention for pediatric patients with postoperative recurrent concealed penis.

**Methods:**

This retrospective longitudinal observational study reviewed 126 pediatric patients who completed a standardized 20-session, pressure-titrated negative-pressure protocol between September 2022 and June 2025 for postoperative recurrence. Primary outcomes included changes in visible penile length (VPL), flaccid penile length (FPL), stretched penile length (SPL), and penile diameter. Morphometric parameters were recorded at baseline, immediately post-treatment, and at a prespecified three-month follow-up.

**Results:**

Significant morphometric gains were achieved across all dimensions (all *P* < 0.001). VPL increased by a mean of 0.76 cm (95% CI: 0.68 to 0.84), demonstrating a substantial clinical effect (Cohen's *dz* = 1.64). Anatomical gains in FPL, SPL, and diameter were similarly robust and successfully maintained at 3 months, with no significant regression (*P* > 0.05). Multivariable analysis revealed that advanced chronological age (*P* = 0.005), higher body mass index (*P* = 0.037), and smaller baseline dimensions (*P* < 0.01) were independent predictors of superior anatomical improvement. The protocol was well tolerated with no serious adverse events.

**Conclusion:**

NPT represents an effective, non-invasive salvage strategy for pediatric recurrent concealed penis. By leveraging mechanotransduction to overcome fibrotic restriction, this modality provides stable anatomical expansion and serves as a viable alternative to complex re-operation, particularly for older children with higher adiposity and more severe initial concealment.

## 1 Introduction

Concealed penis is a pediatric genitourinary anomaly characterized by the obscuration of a morphologically normal phallus by prepubic tissue, dysgenetic dartos fascia, or suprapubic adiposity (1), leading to functional morbidities such as voiding dysfunction and recurrent balanoposthitis (2). Although primary surgical reconstruction via degloving and phallopexy is the established definitive treatment, postoperative recurrence remains a frequent clinical challenge, with literature reporting recurrence rates ranging from 10% to 21.7% following primary correction (3, 4). These recurrences are commonly driven by secondary scar contracture, progressive prepubic adiposity, or inadequate penile base fixation (5–7). Furthermore, these patients frequently present with concurrent weight gain and increased suprapubic fat thickness, factors that directly elevate the risk of secondary surgical failure. Secondary operative reconstruction is technically demanding, as altered anatomical planes and dense postoperative fibrosis significantly elevate the risk of surgical complications and iatrogenic tissue loss (5–7), necessitating the investigation of non-invasive salvage modalities.

Negative-pressure therapy (NPT) offers a mechanobiological alternative to surgical re-intervention by applying controlled suction to induce axial traction and tissue expansion (8). Preclinical models and adult urological applications demonstrate that NPT enhances tissue compliance and downregulates pro-fibrotic pathways in the local microenvironment (9–11). Despite these established mechanisms, empirical data quantifying the morphometric efficacy of NPT as a non-surgical salvage intervention for pediatric recurrent concealed penis are currently absent from the literature.

To address this critical gap, this retrospective pre-post case series evaluates the morphometric outcomes, safety, and three-month durability of a pressure-titrated NPT protocol in pediatric patients with postoperative recurrent concealed penis. The primary objective is to quantify dimensional changes and to identify baseline anthropometric predictors of treatment response.

## 2 Materials and methods

### 2.1 Study population

This retrospective cohort study reviewed the medical records of consecutive pediatric patients (aged 2 to 14 years) who presented with postoperative recurrence of concealed penis and subsequently received negative-pressure therapy between September 2022 and June 2025. Postoperative recurrence was defined as the reappearance of marked concealment with inadequate visible shaft exposure occurring 1 to 2 years after primary surgical reconstruction. Inclusion criteria were the completion of a standardized 20-session treatment course and the availability of complete morphometric penile measurements at baseline and immediately following the 20th session. Exclusion criteria included major concomitant genital anomalies such as hypospadias, endocrine disorders affecting penile development, or missing primary outcome data. As our institution serves as a regional tertiary referral center, the primary surgical approaches prior to recurrence varied. While a subset of patients previously underwent a standardized modified Devine repair with formal penile base fixation at our clinic (12), others were referred from external clinics following diverse or undocumented procedures. In terms of patient allocation, cases with severe anatomical tethering underwent secondary surgical revision, whereas mild recurrences were managed with observation and weight control. The 126 patients included in this study represent cases of moderate-to-severe recurrence whose families actively consented to this negative-pressure salvage protocol.

### 2.2 Intervention protocol

Negative-pressure therapy was administered using a medical vacuum device (Model SW-3501; Sanwe, China) equipped with a soft silicone seal, an adjustable pressure regulator, and an automated fluid-cycling system (Supplementary Figure S1). Each session lasted for 20 min. Treatment was administered once per workday, totaling five sessions per week, until the 20-session course was completed.

An age-stratified, progressive pressure-titration protocol was applied. For patients aged 5 years and under, suction pressure was set at 3 to 4 kPa for sessions 1 to 5, increased to 5 to 6 kPa for sessions 6 to 10, and maintained at 7 to 8 kPa for sessions 11 to 20. For patients older than 5 years, the pressure was initiated at 5 to 7 kPa, increased to 6 to 8 kPa, and maintained at 8 to 10 kPa across the same session intervals. This age-stratified, progressive pressure protocol was designed to accommodate the biomechanical tolerance of pediatric tissues: lower pressures in younger children minimize the risk of microvascular injury, whereas higher pressures in older children provide sufficient mechanical strain to safely overcome dense fibrotic adhesions. Clinicians monitored patients during all procedures. If penile erythema or edema occurred, therapy was temporarily paused until spontaneous resolution, typically within one to three days, before resuming at a retitrated lower pressure.

### 2.3 Outcome measures and measurement procedure

Penile dimensions were recorded at three distinct time points: baseline, post-treatment immediately following the final session of the course, and at a prespecified 3-month follow-up. Four morphometric parameters were evaluated: visible penile length (VPL), flaccid penile length (FPL), stretched penile length (SPL), and penile diameter. To minimize inter-observer variability, all parameters were obtained by a single trained pediatric nurse using a standardized protocol with the patient resting in a supine position. Measurements were performed utilizing a dedicated medical penile ruler and a vernier caliper for mid-shaft diameter. For VPL, the suprapubic soft tissue was fully depressed against the pubic symphysis to minimize confounding by the prepubic fat pad. FPL was recorded without traction. SPL was obtained by applying gentle and firm manual traction along the longitudinal axis. Each parameter was measured two to three times per session, and the mean value was utilized for final statistical analysis.

### 2.4 Statistical analysis

Statistical analyses were conducted using R software version 4.2.1 (R Foundation for Statistical Computing, Vienna, Austria), with continuous variables summarized as the mean ± standard deviation or the median with interquartile range according to the underlying data distribution. Morphometric changes from baseline were evaluated using paired t-tests and Wilcoxon signed-rank tests for sensitivity analysis, with outcomes reported as mean differences, 95% confidence intervals (CIs), and Cohen's *dz* effect sizes to determine the magnitude of clinical improvement. To identify independent predictors of anatomical gain, separate multivariable linear regression models were constructed for each primary outcome, incorporating patient age, body mass index, and baseline values as covariates. Furthermore, potential selection bias stemming from follow-up attrition was assessed by comparing baseline characteristics between participants with and without three-month follow-up data utilizing Welch's *t*-tests. All statistical tests were two-sided, and a *P*-value < 0.05 was considered to indicate statistical significance.

## 3 Results

### 3.1 Baseline patient characteristics

A total of 126 pediatric patients met the inclusion criteria and completed the initial negative-pressure therapy course. The demographic and baseline anthropometric characteristics of the study cohort are summarized in Table 1. The mean age of the patients was 9.24 ± 2.14 years, ranging from 2.50 to 12.92 years. The cohort presented with a mean body mass index (BMI) of 21.41 ± 3.36 kg/m^2^. Pre-treatment morphometric evaluations revealed a mean visible penile length of 1.16 ± 0.54 cm, a flaccid penile length of 2.86 ± 0.54 cm, a stretched penile length of 3.56 ± 0.64 cm, and a penile diameter of 1.29 ± 0.24 cm.

**Table 1:** Baseline characteristics (*N* = 126).

Variable	Mean ± SD	Median (Q1, Q3)	Range (Min–Max)
Age (years)	9.24 ± 2.14	9.46 (8.25, 10.75)	2.50–12.92
BMI (kg/m^2^)	21.41 ± 3.36	20.90 (19.42, 22.98)	14.00–32.20
Weight (kg)	42.50 ± 13.21	41.17 (34.15, 49.50)	15.50–100.00
Height (cm)	139.02 ± 14.35	140.00 (131.00, 148.88)	97.00–176.00

BMI, body mass index; SD, standard deviation.

### 3.2 Immediate post-treatment morphometric outcomes

**Figure 1:**
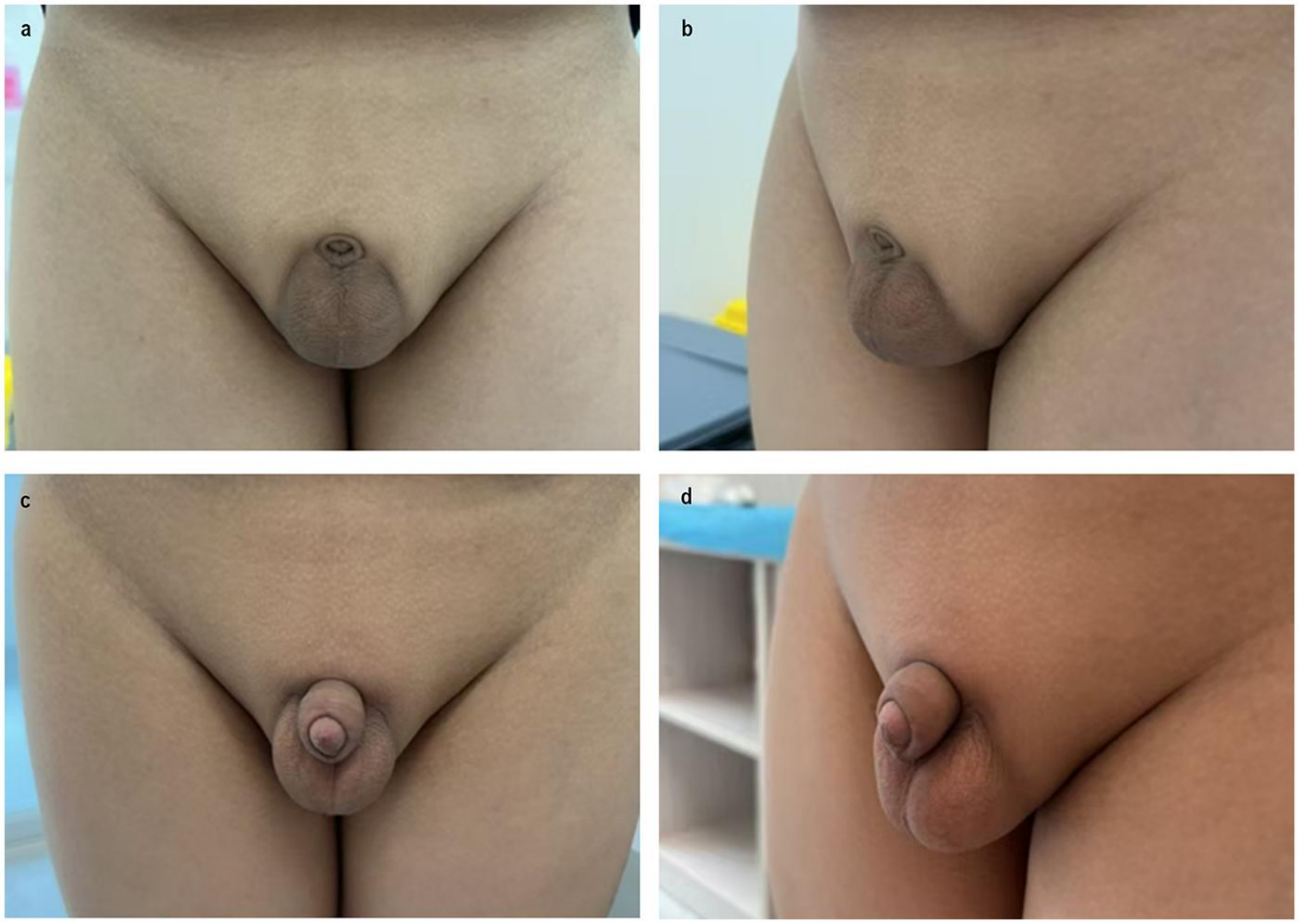
Morphological response to negative-pressure therapy in pediatric recurrent concealed penis. **(a,b)** Pre-treatment baseline with restricted visible shaft and prominent suprapubic adiposity. **(c,d)** Immediate post-treatment anatomy following 20 sessions. Note the expanded visible penile length and complete glans exposure.

Standardized clinical photographs from a representative patient illustrate the treatment response, with an obvious increase in external shaft exposure and clarification of penile anatomy (Figure 1). Following the completion of the prescribed therapy protocol, statistically significant improvements were recorded across all evaluated dimensions (Tables 2, 3). Visible penile length increased by a mean of 0.76 cm (95% CI: 0.68 to 0.84, *P* < 0.001), yielding a large standardized effect size (Cohen's *dz* = 1.64). Similar robust anatomical gains were documented in flaccid penile length with a mean absolute increase of 0.72 cm (95% CI: 0.64 to 0.79, *P* < 0.001), stretched penile length with a mean increase of 0.80 cm (95% CI: 0.72 to 0.87, *P* < 0.001), and penile diameter with a mean increase of 0.22 cm (95% CI: 0.19 to 0.25, *P* < 0.001).

**Table 2:** Penile measurements at baseline and post-treatment.

Outcome	Baseline (*n* = 126)	Post-treatment (*n* = 126)
Visible penile length	1.16 ± 0.54	1.92 ± 0.50
Flaccid penile length	2.86 ± 0.54	3.58 ± 0.59
Stretched penile length	3.56 ± 0.64	4.35 ± 0.70
Penile diameter	1.29 ± 0.24	1.51 ± 0.19

Values are expressed as mean ± SD.

**Table 3:** Morphometric improvements from baseline to 3-month follow-up in the paired cohort (*n* = 64).

Outcome	Baseline	3-month Follow-up	Mean Change	*P*-Value	Cohen's *dz*
Visible penile length (cm)	1.16 ± 0.55	1.79 ± 0.59	0.63	<0.001	1.25
Flaccid penile length (cm)	2.71 ± 0.54	3.38 ± 0.71	0.68	<0.001	1.43
Stretched penile length (cm)	3.34 ± 0.65	4.10 ± 0.78	0.76	<0.001	1.59
Penile diameter (cm)	1.27 ± 0.24	1.44 ± 0.24	0.17	<0.001	0.87

Values are expressed as mean ± SD based exclusively on the 64 patients who completed the follow-up. *P*-values were derived from paired t-tests evaluating the absolute change between baseline and the 3-month follow-up. Cohen's *dz* denotes the standardized mean change (effect size).

### 3.3 Medium-term durability and attrition analysis

A total of 64 patients completed the prespecified three-month follow-up evaluation. To assess potential selection bias resulting from follow-up attrition, a comparative analysis was performed between patients with available follow-up data and those lost to follow-up (Supplementary Table S1). Patients who did not complete the three-month assessment exhibited a slightly higher baseline BMI (22.08 vs. 20.76 kg/m^2^, *P* = 0.027), alongside higher baseline flaccid penile length (3.03 vs. 2.71 cm, *P* < 0.001) and stretched penile length (3.78 vs. 3.34 cm, *P* < 0.001) compared to the returning cohort. Despite this attrition, the morphometric gains achieved immediately post-treatment were successfully sustained at the three-month mark among the returning patients. To strictly eliminate attrition bias, subsequent efficacy evaluations were isolated to the paired subset of 64 patients who completed the follow-up. Direct comparison between baseline and the 3-month mark revealed highly significant, persistent morphological gains across all parameters (Table 3). Importantly, to rule out weight loss as a confounding factor for increased penile exposure, anthropometric changes were analyzed. While absolute body weight and height increased due to physiological growth, the BMI remained remarkably stable throughout the follow-up period (mean change +0.07 kg/m^2^, *P* = 0.595; Supplementary Table S2), confirming that anatomical gains were independent of adiposity reduction.

### 3.4 Predictors of morphometric improvement

Separate multivariable linear regression models were constructed to identify baseline factors independently associated with the magnitude of dimensional improvement, adjusting for age, BMI, and baseline measurements (Table 4). Across all four morphometric domains, a smaller baseline penile dimension emerged as the strongest independent predictor of greater absolute anatomical gain (all *P* < 0.01). Specifically, the model for penile diameter demonstrated the highest explanatory power (*R*^2^ = 0.435). Furthermore, advanced chronological age was independently associated with greater increases in visible penile length (*β* = 0.050, *P* = 0.005). A higher baseline BMI independently predicted larger absolute gains in both flaccid penile length (*β* = 0.024, *P* = 0.037) and penile diameter (*β* = 0.015, *P* < 0.001).

**Table 4:** Multivariable linear regression of factors associated with improvement.

Predictor	*Δ* Visible penile length	*Δ* Flaccid penile length	*Δ* Stretched penile length	*Δ* Penile diameter
Age (per year)	0.050 (0.015, 0.085); 0.005	0.026 (−0.011, 0.064); 0.171	0.025 (−0.014, 0.064); 0.207	0.003 (−0.009, 0.015); 0.613
BMI (per kg/m^2^)	−0.013 (−0.036, 0.010); 0.276	0.024 (0.001, 0.046); 0.037	0.011 (−0.013, 0.034); 0.361	0.015 (0.008, 0.023); <0.001
Baseline value of outcome (cm)	−0.475 (−0.613, −0.337); <0.001	−0.272 (−0.414, −0.131); <0.001	−0.169 (−0.294, −0.044); 0.008	−0.504 (−0.611, −0.397); <0.001
Model R^2^	0.302	0.139	0.063	0.435

Data are *β* coefficients with 95% CIs and *P-*values (*n* = 126). *Δ*, improvement (Post minus baseline).

### 3.5 Safety and tolerability

The therapy was well tolerated by all participants. The most frequent treatment-related observation was transient preputial edema or mild erythema, which occurred in 6 patients (4.76%) during the initial sessions and resolved spontaneously or with minor titration of suction intensity. There were no documented cases of local ecchymosis, blistering, or urinary difficulties. No serious device-related adverse events requiring hospitalization or permanent discontinuation of therapy occurred during the study period.

## 4 Discussion

To our knowledge, this is the largest retrospective study evaluating a standardized NPT protocol as a non-surgical salvage intervention for pediatric recurrent concealed penis. Our findings demonstrate that cyclic negative-pressure mechanotransduction combined with hydro-massage yields significant morphometric improvements across all penile dimensions, with gains successfully maintained at the three-month follow-up. Notably, smaller baseline dimensions, older age, and a higher BMI were identified as independent predictors of greater absolute anatomical improvement. These results provide robust evidence supporting the integration of this non-invasive modality into the clinical management of recurrent concealment.

In patients presenting with postoperative recurrence, the phallus is frequently restricted by dense fibrotic adhesions and secondary scar contracture (7). While surgical re-intervention carries elevated risks of persistent lymphedema and recurrent retraction, NPT utilizes continuous mechanotransduction to address these fibrotic barriers without invasive dissection (13). As established in distraction histogenesis models, external negative pressure induces critical macroscopic and microscopic deformations within the tissue bed (14). This mechanical strain activates mechanosensitive signaling pathways in local fibroblasts, leading to the downregulation of pro-fibrotic collagen deposition and the reorganization of the extracellular matrix (15, 16). Furthermore, cyclic negative pressure enhances microvascular permeability and stimulates neoangiogenesis, which optimizes the local microenvironment for sustained anatomical expansion (17). Consequently, NPT effectively softens restrictive cicatricial tissue at the penile root, providing a robust biomechanical explanation for the observed morphological gains.

Our findings align with adult penile rehabilitation studies using vacuum erectile devices (VED) (8–10). However, while adult VED protocols emphasize antihypoxic mechanisms to prevent smooth muscle apoptosis (8, 10), our pediatric data indicates that NPT primarily acts via mechanical disruption of secondary scar contractures at the penile base. Despite differing mechanistic targets, both demonstrate the efficacy of cyclic negative pressure for stable anatomical expansion (13).

A pivotal finding of this study is that advanced chronological age, higher BMI, and smaller baseline dimensions independently predict the magnitude of absolute anatomical improvement. The positive correlation with age may be partly attributable to improved behavioral compliance and a more reliable vacuum seal in older children, enabling more consistent delivery of negative-pressure-mediated mechanical loading. This optimal loading maximizes downstream antihypoxic and antifibrotic effects (9). Furthermore, the pronounced efficacy observed in patients with higher BMI may reflect a larger adiposity-driven concealment component; suprapubic and prepubic soft tissue is a recognized contributor to the buried penis phenotype, and pediatric series have reported recurrence clustering in obese boys after surgical repair (4, 18). In this setting, the applicator may function as a temporary spacer that displaces suprapubic soft tissue and increases exposure of the corporal bodies. Finally, the inverse association between baseline dimensions and gain is consistent with the established mechanobiology of stretch-induced growth. Under comparable loads, more severely restricted tissues can experience proportionally greater strain, which may amplify mechanotransduction pathways implicated in cellular proliferation and extracellular matrix remodeling (14, 19, 20). Furthermore, while immediate post-treatment increases in penile diameter may partially reflect transient vascular engorgement and interstitial edema, the persistence of significant diametric expansion observed at the 3-month follow-up suggests true macroscopic morphological remodeling rather than acute congestion.

Several limitations warrant consideration. First, the retrospective, single-center design introduces selection bias and limits generalizability. As our institution functions as a tertiary referral center, calculating an exact regional recurrence rate or establishing a precise denominator for initial surgeries was unfeasible. Second, although intra-observer variability was mitigated by averaging multiple measurements, the evaluating nurse was not blinded to the treatment time points, introducing potential measurement bias. Third, lacking a randomized control arm precludes definitive causal comparisons. Furthermore, this single-arm design and limited sample size prevented a direct efficacy comparison between obese and non-obese subgroups. Larger comparative trials are necessary to confirm NPT's specific salvage advantages in obese patients. Fourth, the substantial attrition rate (49% lost to follow-up at 3 months) introduces significant survivorship bias, necessitating a cautious interpretation of the medium-term durability data. Finally, the three-month follow-up offers only medium-term insights. Future multi-center, observer-blinded prospective trials extending through puberty are required to ascertain the long-term durability of this tissue remodeling.

## 5 Conclusion

NPT represents an effective, non-invasive salvage strategy for pediatric recurrent concealed penis, yielding stable anatomical improvements through cyclic mechanotransduction. Optimal absolute gains are specifically associated with advanced chronological age, higher BMI, and smaller baseline penile dimensions. Given its safety and the technical complexity of reoperation, NPT offers a viable alternative for managing this challenging clinical scenario. Future multi-center trials are warranted to establish long-term clinical guidelines.

## Data Availability

The raw data supporting the conclusions of this article will be made available by the authors, without undue reservation.
