# Supplementary material for: Efficacy and anthropometric predictors of negative-pressure therapy for recurrent concealed penis in pediatric patients
Source: Front Pediatr. 2026 Jun 18;14:1845192. doi: 10.3389/fped.2026.1845192 (PMC13323223; doi:10.3389/fped.2026.1845192)
Supplement: Supplementary file 1 [file Table1.docx]

Supplementary Material

# Supplementary Data

**Supplementary Table S1**. Baseline characteristics by follow-up availability.

| Variable | Follow-up Available (n=64) | Lost to Follow-up (n=62) | P Value |
| --- | --- | --- | --- |
| Age (years) | 9.09 ± 2.37 | 9.40 ± 1.88 | 0.422 |
| BMI (kg/m²) | 20.76 ± 3.51 | 22.08 ± 3.08 | 0.027 |
| Baseline VPL (cm) | 1.16 ± 0.55 | 1.15 ± 0.53 | 0.988 |
| Baseline FPL (cm) | 2.71 ± 0.54 | 3.03 ± 0.50 | <0.001 |
| Baseline SPL (cm) | 3.34 ± 0.65 | 3.78 ± 0.55 | <0.001 |
| Baseline diameter (cm) | 1.27 ± 0.24 | 1.31 ± 0.23 | 0.295 |

Values are mean ± SD. *P-*values from Welch’s t-tests. VPL, visible penile length; FPL, flaccid penile length; SPL, stretched penile length.

**Supplementary Table S2**. Anthropometric changes during the 3-month follow-up period in the paired cohort (n = 64).

| Anthropometric Parameter | Baseline | 3-month Follow-up | Mean Change | P-Value |
| --- | --- | --- | --- | --- |
| Body Height (cm) | 137.95 ± 16.38 | 141.48 ± 15.61 | 3.54 | < 0.001 |
| Body Weight (kg) | 41.06 ± 15.36 | 43.22 ± 15.64 | 2.16 | < 0.001 |
| Body Mass Index (BMI, kg/m²) | 20.76 ± 3.51 | 20.82 ± 3.64 | 0.07 | 0.595 |

Values are expressed as mean ± SD based exclusively on the 64 patients who completed the follow-up. While absolute body height and weight increased significantly due to normal physiological growth during the three-month interval (both *P* < 0.001), the BMI remained highly stable (P = 0.595). This confirms the absence of natural weight loss or adiposity reduction as a confounding factor for the observed penile morphometric gains.


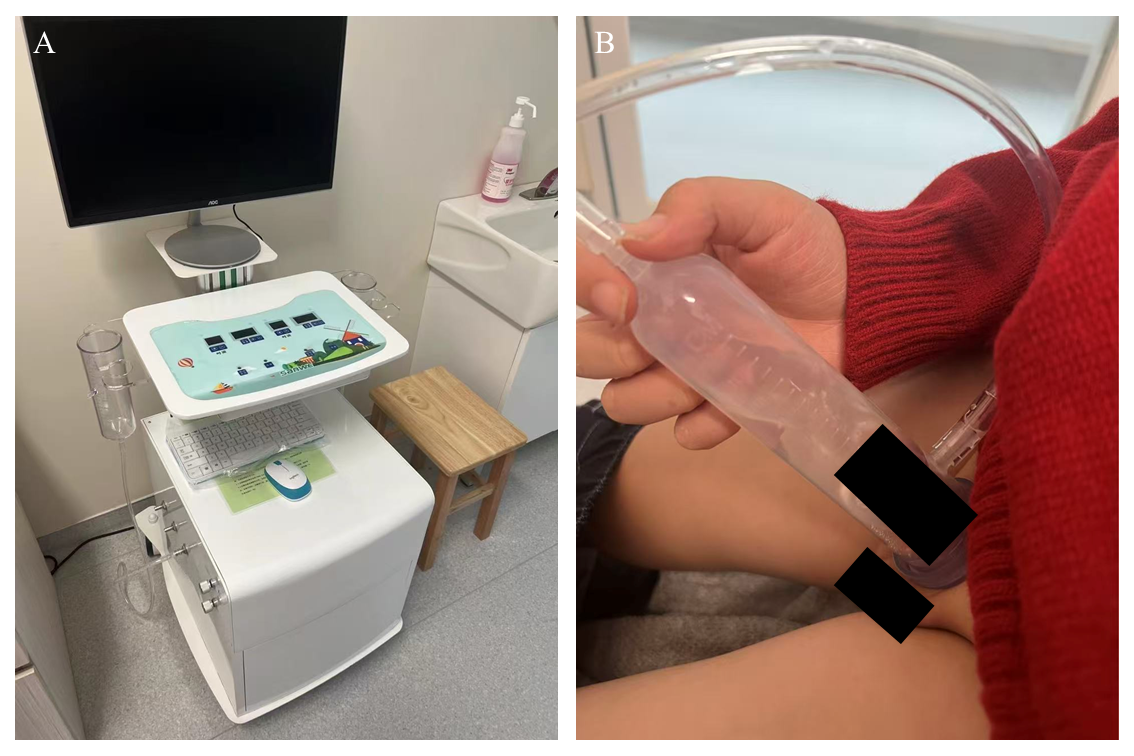


**Supplementary Figure S1.** Negative-pressure therapy device and clinical application.

(A) The SW-3501 medical vacuum device (Sanwe, China) used in this study; (B) Clinical application: the silicone applicator is positioned against the pubic symphysis to deliver controlled cyclic negative pressure, for scar contracture release and tissue expansion.
